# A long-term retrospective study on sporadic Burkitt lymphoma in chinese population

**DOI:** 10.1097/MD.0000000000018438

**Published:** 2020-01-31

**Authors:** Xiaoyun Yang, Qianru Huang, An Li, Yuan Chen, Wei Xu, Jianyong Li, Yaping Wang, Yongjun Fang

**Affiliations:** a Department of Hematology and Oncology, Children's Hospital of Nanjing Medical University; b Key Laboratory of Hematology, Nanjing Medical University; c Jiangsu Province Hospital, The First Affiliated Hospital of Nanjing Medical University; d Department of Otorhinolaryngology, The Second Affiliated Hospital of Nanjing Medical University, Nanjing, China.

**Keywords:** adolescents and adults, Burkitt lymphoma, children, prognosis, retrospective study

## Abstract

Burkitt lymphoma (BL), an aggressive malignancy, brings a prognosis varying among children, adolescents, and adults. Most of previous retrospective studies of BL focused on a part of population. This study aimed to find the leading prognostic factors in BL among patients of different age groups. World Health Organization classification of lymphoid neoplasms in 2008 and revision in 2016 were used as diagnostic criteria for BL. We compared the laboratory results and clinical manifestations in 2 age groups by Kaplan–Meier survival analysis. Our study strongly indicated that age >14 years and lactate dehydrogenase >570 U/L were 2 powerful prognostic factors for BL. The results indicated that poor prognosis may be for the poor tolerance and low dose of drugs in adolescents and adults.

## 1 Introduction

Burkitt lymphoma (BL), a kind of non-Hodgkin lymphoma (NHL) initially reported by Donnis Burkitt in 1958,^[1]^ features hyperinvasiveness, rapid clinical progress, and high lethality. Mostly, BL originated from B cells in the follicular germinal center (GC). BL is characterized by diffuse tumor cells surrounded by some normal phagocytic cells, which is called “starry sky” phenomenon. Sporadic BL (sBL) attacks regions outside Africa, with an incidence of about 4 per million in the United States,^[2]^ and its clinical features change with race and geology. In 1975, chromosomal aberration, described as translocation of the distal region of the long arm of chromosome 14 with the long arm of chromosome 8, was first discovered in the development of BL.^[3]^ Then, *MYC* gene was confirmed as the break point of t(8;14) translocation. As the research continued, the scholars realized the *MYC* gene interacted with immunoglobulin (IG) heavy-chain locus.^[4,5]^ Till now, it is considered that the abnormal expression of MYC gene, mostly triggered by t(8;14)(q24;q32) translocation, is a mechanism behind BL.^[6,7]^ C-myc is a proto-oncogene at 8q24 and IgH is an immunoglobulin heavy chain gene at 14q32. t(8;14)(q24;q32) is translocation by C-myc and IgH resulting in abnormal regulation of C-myc gene and high expression. Furthermore, dysregulation of some signaling pathway protein phosphorylation may account for one of pathogenesis of hematological malignancies. For example, granulocyte colony-stimulating factor receptor (G-CSFR) can trigger the differentiation from bone marrow progenitor cells to granulocytes. Among chronic neutrophilic leukemia, myelodysplastic syndrome, acute myeloid leukemia, and atypical chronic myelogenous leukemia, G-CSFR was usually with aberrances like specific amino acid phosphorylation.^[8–10]^ Researchers have reported similar achievements that overexpression and phosphorylation of PAG1 could destroy the balance in B-cell receptor (BCR) signaling pathway in BL. Also, serine 194 phosphorylated Fas-associated death domain protein may be related to the proliferation of neoplastic B cells in BL.^[11,12]^

According to the literature, sBL in North America and Europe makes up to 30% to 40% of children NHL and 1% to 5% of adults NHL, respectively.^[13]^ Obviously, these proportions show marked difference. However, the reason has not been found out. Here, we established a survey to explore this reason using 2 age groups (53 cases in children group and 29 cases in adolescents and adult group).

## 2 Materials and methods

### 2.1 Patients

Patients newly diagnosed with BL were eligible. Between January 1, 2010 and September 30, 2018, the study included 53 patients (≤14 years) diagnosed at Children's Hospital of Nanjing Medical University versus 29 patients (>14 years) diagnosed at Jiangsu Province Hospital. The patients were all Han Chinese and had no genetic correlation with each other. The final diagnosis depended on the pathological examination, immunohistochemistry, and clinical manifestations. World Health Organization classification of lymphoid neoplasms in 2008 and revision in 2016 were used as diagnostic criteria.^[14,15]^ Pathological biopsy specimens and bone marrow (BM) biopsy specimens displayed poorly differentiated lymphoma cells showing a medium size and nuclear fission scattered among the cytoplasmic light-stained macrophages, forming a starry image. Relevant examinations consisted of immunohistochemistry, fluorescence in situ hybridization (FISH), chromosomal karyotype, and flow cytometry. Immunohistochemistry was performed to test the expression of B cell–associated antigens (CD19, CD20, CD22 and CD79a), level of Ki-67, expression of *Bcl-6*, *Bcl-2*, as well as *C-myc*. Level of Ki-67 indicated the proliferation rate of tumor cells. *Bcl-6*, *Bcl-2*, and *C-myc* worked as proto-oncogenes. Most of BL patients showed positive expression of *Bcl-6* and *C-myc* and negative expression of *Bcl-2*. With effusions, flow cytometry was introduced for detection of B cell–associated antigens. Also, FISH examination was used to check whether abnormal t(8;14) existed or not. Leukemia was diagnosed with >20% of abnormal naive lymphocytes on BM smear. Excluded were those who had developed other hematological disorders, cancers, or HIV, or lost most of the treatment-related information, or been misdiagnosed.

The study protocol was reviewed by the Medical Ethics Committee of Children's Hospital of Nanjing Medical University and the Medical Ethics Committee of Jiangsu Province Hospital. Informed consents were obtained.

### 2.2 Staging and stratification of treatment

The disease was staged according to St. Jude staging system for children and Ann Arbor staging system for adults (Table 1). Pretreatment evaluations included physical examination, assessment of symptom group B, peripheral blood cell analysis, liver function, renal function, electrolyte, lactate dehydrogenase (LDH) level, blood coagulation function, Epstein-virus (EBV) antibody level, EBV-DNA, hepatitis B virus (HBV) detection, human immunodeficiency virus (HIV) detection, BM biopsy, cerebrospinal fluid analyses, ultrasound, x-ray, computed tomography (CT), PET/CT (for all adults and some children), magnetic resonance imaging (MRI), and skeletal scintigraphy. The normal ranges of biochemical indexes set by the 2 hospitals were consistent. Treatment for children was designed according to the 4 risk grades (Table 2). Treatment for adolescents with adults was designed according to the risk grades compiled by National Comprehensive Cancer Network (NCCN) guidelines. Low risk grade indicated the patients’ normal LDH level, complete excision of abdominal lesions, or the presence of a single external abdominal lesions <10 cm in diameter. Apart from patients in low-risk grade, the remaining patients were classified in high-risk grade.

**Table 1:**
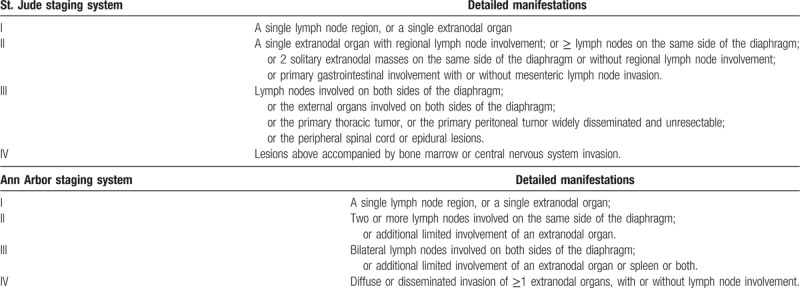
Criteria for disease stages. St. Jude staging system for children and Ann Arbor staging system for adolescents and adults.

**Table 2:**
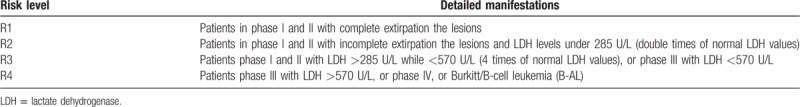
Treatment for children was based on the 4 levels.

### 2.3 Therapy and evaluation

**Figure 1:**
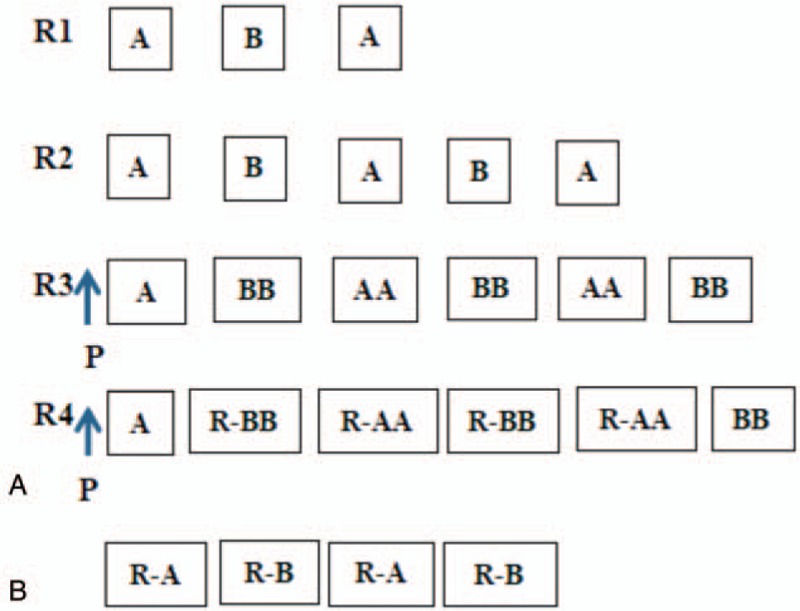
Treatment for Burkitt lymphoma. (A) CCCG-BNHL-2009 and CCCG-BNHL-2015 schemes for children. A regimen contains CTX, VCR, ADR, Ara-C, and prednisone. B regimen contains of ifosfamide, VP-16, MTX, VCR, and prednisone. P: inductive treatment. R: Rituximab. (B) Hyper-CVAD schemes for most adolesents and adults. A regimen contains of CTX, ADR, VCR, and DXM. B regimen contains of MTX, calcium folinate, and Ara-C.

In this study, all the children under 14 years received chemotherapy according to Chinese Children’ s Cancer Group-B cell non-Hodgkin lymphoma-2009 (CCCG-BNHL-2009) protocol and CCCG-BNHL-2015 protocol. Then, hyper fractionated cyclophosphamide, vincristine, doxorubicin and dexamethasone (hyper-CVAD) protocol, the most common for BL, was implemented in our adolescents with adults. Every protocol contains A and B regimens with difference in dose and compositions. Conventional rituximab was given in adolescents with adults group, but in children group, only patients at R4 grade were required to receive rituximab and those at the other 3 grades received rituximab as they will. Intrathecal injection was administered in case of central invasion. Information of routine antineoplastic use was detailed in Figure 1.

Due to poor effect of first-line regimen or cost restriction, some adolescents with adult patients were treated with other second-line regimens, including DA-EDOCH, VCDP.

### 2.4 Follow-up Visit

Patients were followed up through outpatient interview and telephone. The follow-up data of all 53 children and 29 adolescents with adults were eligible. The follow-up ended at September 30, 2018. Survival time was calculated by the time from final diagnosis to death or final follow-up time.

### 2.5 Statistical analysis

Statistical analysis was carried out by SPSS 20.0 (SPSS Inc, Chicago, IL). *χ*^2^ test and Fisher exact probability method were used to evaluate statistical differences in demographic and clinical data. Kaplan–Meier survival analysis was used to evaluate the survival rate, Log-Rank test was performed for univariate prognostic analysis, and COX proportional risk regression model for multivariate prognostic analysis. P value <.05 was considered significant.

## 3 Results

### 3.1 Patients

A total of 53 children and 29 adults (63 males, 76.83%; 19 females, 23.17%; a ratio of 3.32:1) were finalized into the research (Table 3). Based on 2 staging systems, the number of patients at stage I and II was 17 (20.73%), and that at stage III and IV was 65 (79.27%). Obviously, the patients were mainly at stage III and IV despite of their age.

**Table 3:**
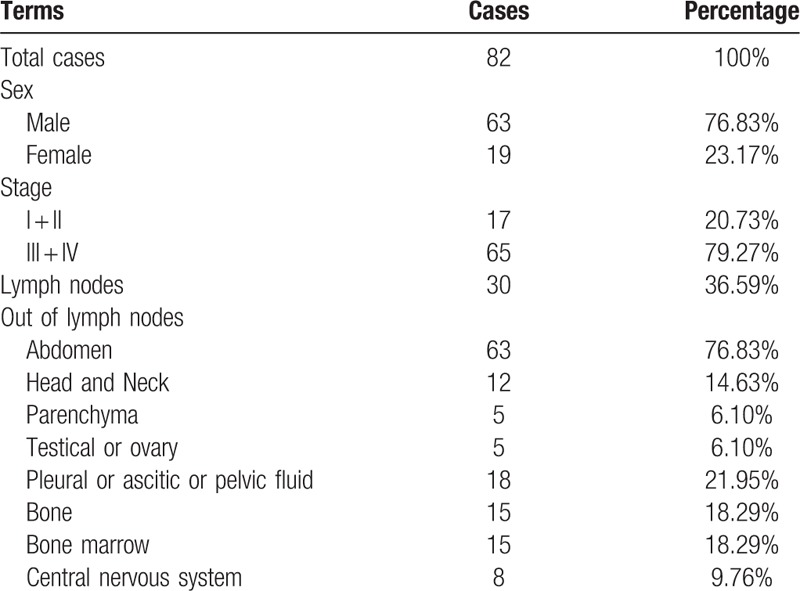
Features of all enrolled patients. Items contained sex, stage, and involvements regions.

The children group included 41 males and 12 females (ratio 3.42:1, median age 5 years, range 1 year 9 months to 13 years 3 months). The adolescents and adults group included 22 males and 7 females (ratio 3.14:1, median age 30 years, rang 17 years 5 months to 67 years). Sex ratio showed no statistically significant difference (*χ*^2^ = 0.024, *P* = .878) (Table 4).

**Table 4:**
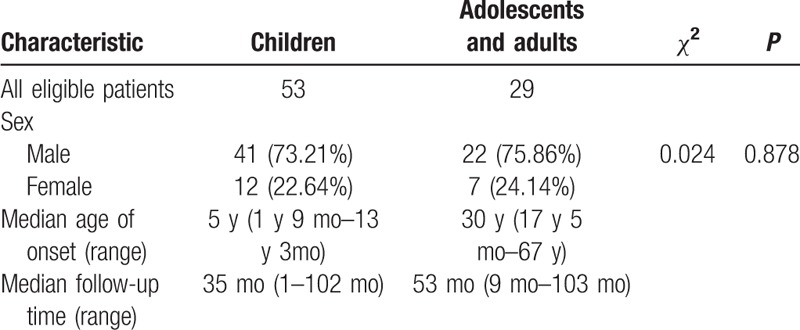
Characteristics of sex, median age, median follow-up between children group and adolescents/adults group.

Table 5 showed the distribution of children, and adolescents and adults at different stages. On the proportions of patients at stage III (*P* = 0.03) and IV (*P* = 0.002) showed difference between groups. Tabular displayed a majority of children at stage III and IV, and adolescents with adults at stage IV (Table 5).

**Table 5:**
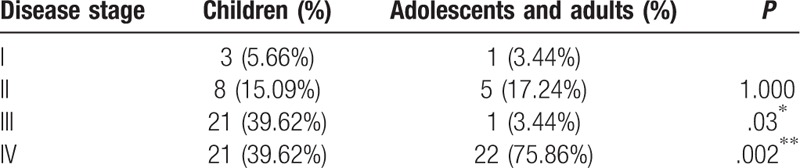
Component proportion ratio on disease stage in 2 groups.

### 3.2 Clinical Features

BL involvement was evaluated. Patients presented accessible masses in abdominal regions or head and neck before the first hospital visit (Table 3). Primary abdominal involvement (63/82, 76.83%) was mainly manifested by abdominal mass, pain, bloating, diarrhea, vomiting, gastrointestinal bleeding, and many more; head and neck involvement (12/82, 14.63%) by difficulty breathing, swallowing, nasal congestion; lymph node involvement (30/82, 36.59%) by local masses, pain, fever, ulceration; fluid involvement (18/82, 21.95%) (If the quantity of fluid was large enough, the patients presented difficulty breathing, pain, bloating, among others); BM involvement (15/82, 18.29%) by fever, dizziness, weakness, pale complexion, petechiae, bleeding, among others; bone involvement (15/82, 18.29%) by pain, restricted movement, joint rigidity, among others; and central nervous system involvement (CNS, 8/82, 9.76%) by head ache, dizziness, numbness of limbs, back pain, vomiting, ghosting, among others. Involvement also appeared in parenchyma (5/82, 6.1%) and testis or ovary (5/82, 6.1%).

In children group, the 3 most involved regions were intestine (26/53, 49.06%), lymph nodes (19/53, 35.85%), and pleural or ascitic or pelvic fluid (13/53, 24.53%). Through the table of aggregate information (as shown by Table 6), the abdominal involvement (35/53, 66.04%) dominated in children group.

**Table 6:**
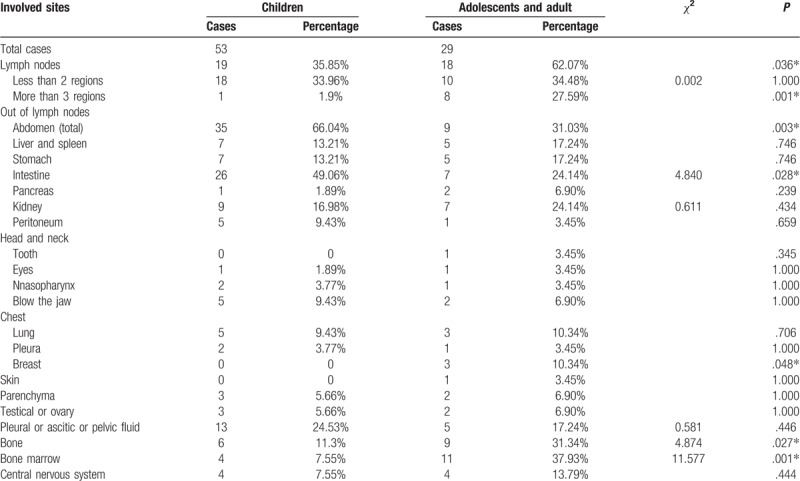
Involved regions of 2 groups.

In adolescents with adults group, the top 3 involved regions were lymph nodes (18/29, 62.07%), BM (11/29, 37.93%), and bone (9/29, 31.34%). As shown by Table 6, the lymph node involvement dominated in adolescents with adults group. All the involved regions in 2 groups were summarized in Table 6.

We applied *χ*^2^ test and Fisher exact test. The results revealed significant difference between 2 groups. The statistically significance of involvements mainly focused on lymph node regions (*P* = .036) especially >3 infiltrated regional lymph nodes (*P* = .001), abdomen (*P* = .003) especially in intestine (*P* = .028), breast (*P* =.048), bone (*P* = .027), and BM (*P* = .001).

### 3.3 Clinical examination analysis

Here, we mainly focused on 2 stages for analysis, as the patients mainly in stage III and stage IV. Immunohistochemical results for phase III and phase IV patients were summarized in Table 7. First, *Bcl-2* level (*P* = .026) and Ki-67 level (*P* = .006) showed difference between 2 groups. The number of patients with a Ki-67 level >95% was larger in children group than that in adult group. LDH level also showed meaningful difference (*P* = .189), indicating more children had high level of LDH than adolescents and adults. Besides, the serum ferritin level presented obvious difference between 2 groups (*P* = .047). More adolescents with adults had a high level of ferritin than children. The positive rate of EBV infection in all the patients reached 21.43% (6/28) according to peripheral blood EBV-DNA test.

**Table 7:**
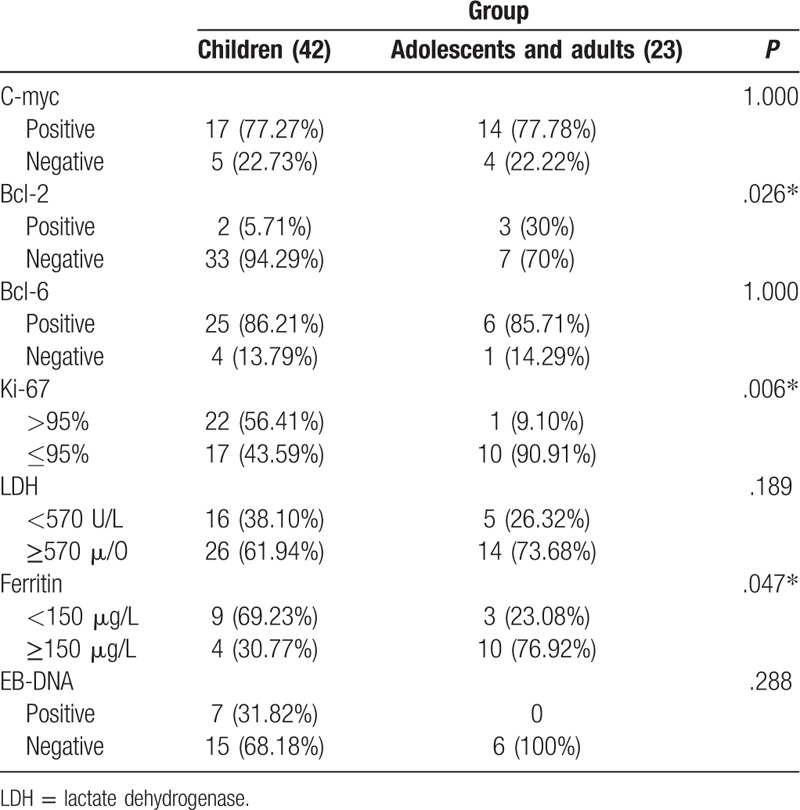
Summary of BL relevant characteristic indicators in III and IV stages.

### 3.4 Follow-up and survival

#### 3.4.1 Overall survival

**Figure 2:**
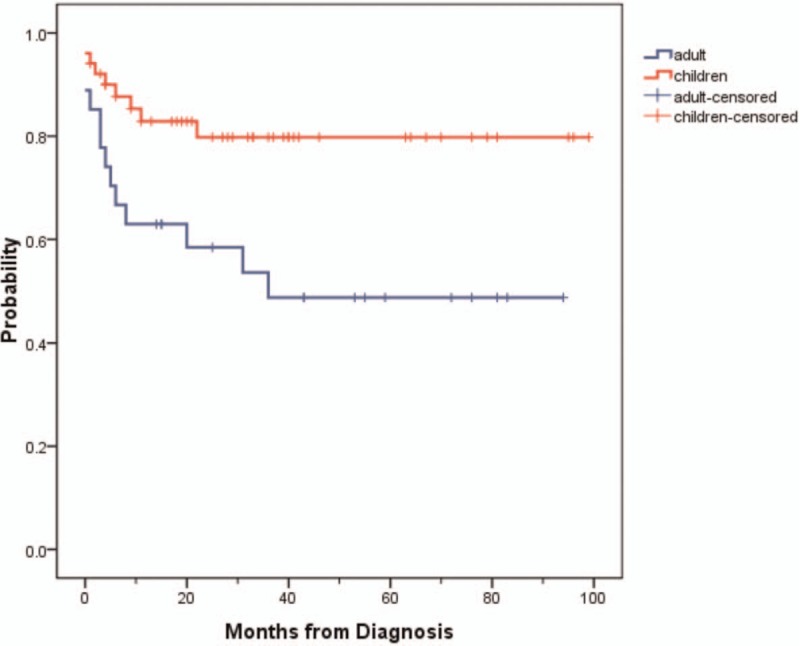
Five-year overall survival (OS) rate for children and adolescents/adults groups. The OS rates in 2 groups showed significance (*P* = .010).

At the end of follow-up, there were 43 cases alive in children group and 16 cases alive in adolescents with adults group. The total 5-year overall survival (OS) rate for 2 groups was 67.6%. The OS rates in 2 groups showed significance (*P* = .010) (Fig. 2).

In children group, 6 (60%) cases died of tumor relapse or progression, 3 (30%) of tumor lysis syndrome (TLS), and 1 (10%) of ambiguous reasons. The median survival time and average survival time were about 20 months and 31.3 months, respectively. The 5-year OS rate in children was 80%.

In adolescents and adults, 6 (46.15%) cases died of tumor relapse or progression, 2 (15.38%) of TLS, and 5 (38.46%) of intracranial hemorrhage, severe infections, and other causes. The median survival time and average survival time were about 25 months and 30.7 months, respectively. The 5-year OS rate in adolescents and adults was 49%.

#### 3.4.2 Univariate analysis of prognostic factors

**Figure 3:**
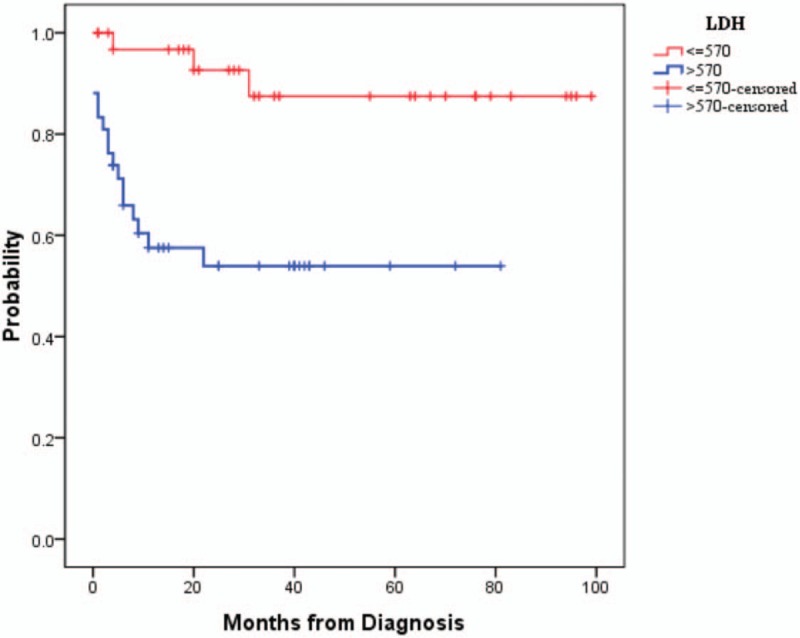
The survival curves in 2 groups were drafted by Kaplan–Meier analysis. Only LDH level showed statistical difference among patients (*P* = .001). LDH = lactate dehydrogenase.

Log rank statistical analysis was used to analyze prognostic factors, including sex, stage, *C-myc, Bcl-2, Bcl-6*, Ki-67, LDH level, BM infiltration and CNS involvement. As a whole, the stage of disease (*P* = .037), BM involvement (*P* = .013), CNS involvement (*P* = .037) showed significance. The results indicated that BM (*P* = .002) and CNS involvement (*P* = .014) had significant effect on OS in children. Besides, the stage of disease (*P* = .022) still had effect on OS in adolescents and adults. Other datasets showed no statistical difference. The survival curves in 2 groups were drafted by Kaplan-Meier analysis. Only LDH level showed statistical difference among patients (Fig. 3).

#### 3.4.3 Multivariate analysis on prognostic factors

Multivariate analysis on prognostic factors was carried out through Cox proportional hazard model. Factors included age, stage, *C-myc, Bcl-6,* LDH, BM involvement, and CNS involvement. The results suggested that age, CNS involvement, and BM involvement were the independent risk factors for prognosis.

## 4 Discussions

Herein, our study reported the clinical features of 53 children and 29 adolescents with adults with sBL. Most of the patients entered stage IV and the incidence of stage IV sBL was much higher in children, indicating that adolescents and adults are more likely to suffer a poor prognosis. The abdominal involvement dominated children group, whereas lymph node dominated the adolescents and adults group. The reasons may be that nearly 80% of patients in 2 groups showed different disease stage and tolerance. More interestingly, we found a proportion of children with the first symptom of intussusception, suggesting that intussusception should also be listed into the exclusion criteria.

In adolescent and adult patients at III and IV stages, the low positive rate of *Bcl-2* prompted us to confirm whether the primary diagnosis was correct or not. It has been reported that tumor cell growth cannot be evaluated by a single indicator.^[16,17]^ We finally realized that all of the 5 patients were tested *C-myc* gene-positive and the level of Ki-67 was close to 100%. These results support the diagnosis of BL. At same time, lower or weakly positive rate of *Bcl-2* (<20%) could support the diagnosis of BL, which is tally with the results of our study (6/43, 13.95%).^[18]^ Apart from *Bcl-2*, the proportion of patients with a Ki-67 level ≤95% (10 cases) was lower in adolescents and adults than that in children. A convention idea is that Ki-67 level is proportional to the proliferation activity of tumor cells and a higher Ki-67 level means a shorter OS of a patient with mantle cell lymphoma.^[17,19]^ But in our study, adolescents and adults with a lower Ki-67 level faced a worse prognosis than that of children.

In this study, the positive detection rate of EBV was 22.73%, close to the level reported in China and lower than that in other countries.^[20,21]^ Usually, EBV infection was considered as one of pathogenesis of regional BL. The results indicated that the pathogenesis of sBL was not related to EBV, but this indication should be supported by more data.

Difference was found in the clinical sBL features, including race, territory, and environment. However, retrospective studies on BL have not been much reported. A report in America showed that sBL mostly occurred in children of 3 to 8 years, with an average onset age of 7.8 years; BL mostly occurred in male children and the male:female ratio was 3.9:1; lymph nodes and abdomen were the 2 most involved sites; the percentage of acute lymphoblastic leukemia L3 was 14%.^[13]^ Another report in Taiwan covered 17 children and 14 adults with a male:female ratio of 1.6:1; age of onset ranged from 0 to 70 years and the median age was 13 years; nearly 50 percentage of all patients were in middle and last stage.^[22]^ A report in Sichuan province in China revealed that the disease was more likely to attack males, with a male:female ratio was 9.75:1; nearly one-third of patients were in I and II stage; head and neck and superficial lymph nodes were involved more often. The expression of *Bcl-6* was nearly 93%; Ki-67 level exceeded 90%; expression of *Bcl-2* was all negative; positive rate of *C-myc* gene was 76.7%.^[23]^ Bcl-2 worked as an oncogene encoding an integral outer mitochondrial membrane protein that decreases the rate of apoptosis,^[24]^ and it was always positive among 50% to 90% of patients with diffuse large B-cell lymphoma.^[25]^

Controversy still exists on what can indicate a poor BL prognosis, like old age, high IPI score, tumor diameter of >10 cm, high LDH level, albumin decrease, higher clinical stage, BM, and CNS invasion. Given the low incidence of BL, lack of prospective studies and differences in clinical characteristics, the related study results vary. Our study indicated that age >14 years, BM involvement, and CNS involvement were powerful indicators. However, only CNS involvement and BM involvement were independent risk factors. Usually, increased levels of LDH suggested that patients have high tumor burden, extranodal metastases, and high degree of malignancy. So, LDH was significantly increased in blood or body fluids near tumor tissue. In our study, LDH level was not the most significant difference between children and adolescents/adults group; however, it seemed LDH level has statistical difference in survival rate. This may be related to the fact that we only count the LDH difference between phase III and phase IV patients, and all the patients in the survival analysis. It may indirectly indicate that LDH was an indicator that can be used for both children group and adolescents/adults group to guide long-term survival.

Furthermore, different therapies may account for prognosis among 2 groups partly. In this study, all the children under 14 years were treated by chemotherapy according to CCCG-BNHL-2009 protocol and CCCG-BNHL-2015 protocol. Then, hyper-CVAD protocol was carried out in our adolescent with adult patients. Obviously, children group had higher chemotherapy intensity than adolescents/adults group. Also, individualized use of rituximab may reduce side effects, so that we can achieve high OS rate in children group.

sBL is a kind of malignant disease. As shown by our study, the OS of adolescent with adult sBL patients was worse than children patients. Some clinical index may indicate the correlation with pathogenesis of sBL, such as region involvements. Also, regimen intensity may be another reason, which contributes to survival differences between the groups. More prospective studies are needed to explore targets for improving therapeutic outcomes.

## Acknowledgments

The authors thank the pediatric research institute affiliated to Nanjing Medical University for experiments.

## Author contributions

**Data curation:** Xiaoyun Yang, Qianru Huang.

**Formal analysis:** Yaping Wang, Yuan Chen.

**Funding acquisition:** Yongjun Fang.

**Investigation:** An Li.

**Project administration:** Jianyong Li.

**Resources:** Wei Xu, Jianyong Li.

**Writing – original draft:** Xiaoyun Yang.

**Writing – review & editing:** Yongjun Fang.
